# Another Perspective on Cannabis and Emergency Medicine in Colorado

**DOI:** 10.5811/westjem.2019.8.44882

**Published:** 2019-10-16

**Authors:** Kennon Heard, Andrew A. Monte, George Sam Wang

**Affiliations:** * University of Colorado School of Medicine, Department of Emergency Medicine, Section of Medical Pharmacology and Toxicology, Aurora, Colorado; † Rocky Mountain Poison and Drug Center, Denver, Colorado; ‡ University of Colorado School of Medicine, Department of Pediatrics, Section of Pediatric Emergency Medicine, Aurora, Colorado

This editorial is written in response to Roberts BA. Legalized Cannabis in Colorado Emergency Departments: A Cautionary Review of Negative Health and Safety Effects. *West J Emerg Med*. 2019;20(4):557–72. ( https://escholarship.org/uc/item/6xb8q31x)

Dr. Roberts has delivered an excellent review of many medical aspects of cannabis use and the effect of cannabis legalization on emergency medicine in Colorado.^1^ As emergency physician researchers in Colorado, we echo many of his concerns. As he notes, since legalization, we have identified an increase in accidental pediatric exposures (some of which resulted in severe effects)^2^–^4^, an increase in emergency department visits for hyperemesis (most likely related to cannabinoid hyperemesis),^5^ an increased number of visits attributable to cannabis edibles,^6^ a disproportionate increase in adult^7^ and adolescent^8^ mental health visits related to cannabis, and an increased number of visits for cannabis toxicity (greater in tourists than locals).^9^ These effects are measurable, and while the direct attribution of these changes to cannabis legalization are limited to observational data that is subject to temporal trends, selection bias, and confounding, we believe the links between these changes and cannabis legalization are plausible, consistent and relevant.

While much of the focus in Colorado has been on recreational cannabis, it is important to note that many of the issues identified began before recreational cannabis was available in 2014. In Colorado, medical cannabis was legalized in 2000 and has been widely available since 2009. In Colorado, the qualifying medical conditions for cannabis use include the following: cancer, glaucoma HIV, severe pain, seizures, nausea, muscle spasm, post-traumatic stress disorder (PTSD), autism spectrum disorder, and cachexia.^10^ As of June 2019, almost 84,000 patients have an active medical marijuana registration, 337 (0.4%) less than 18 years of age.^11^ As with any therapy, the adverse effects we have identified must be balanced against the potential benefits to patients and society. However, there are few high-quality evidenced based studies to support these recommendations. Without clinical trials the measurement of the positive effects of cannabis remain largely anecdotal. There are additional concerns for reported cannabinoid content and claims on treatment for disease. The United States Food and Drug Administration (FDA) has issued numerous warning letters to various cannabidiol manufacturers for false claims in relation to disease diagnosis and treatment.^13^ The medical utility of cannabis is further limited by insufficient training provided to medical professionals and trainees, in addition to the reliance of many users on non-medical providers to guide therapeutic choices. For example, many dispensaries will recommend cannabis to pregnant women despite various national guidelines cautioning against this practice.^12^ The medical benefits of cannabis should have been evaluated using accepted clinical standards prior to providing legal status as medical treatments.

Recreational use has no demonstrated inherent health benefit. While some have suggested that it may increase relaxation and reduce stress, there are no clinical studies to support those claims. One plausible health benefit is the substitution of cannabis for other more dangerous recreational drugs; however, this is also not studied. Unfortunately, in Colorado we see that cannabis is also often combined with alcohol and other drugs and the relative increase in adverse effects may outweigh this potential benefit. Despite the observed increase in cannabis related driving fatalities in Colorado, 55% of cannabis users believed it was safe to drive under the influence of cannabis.^14^ There have been mixed results on how marijuana legalization has affected medical and nonmedical opioid use and prescribing.^15^–^16^

The discussion around the impact of cannabis on the healthcare system is (as with many issues) not absolute. When we speak to cannabis supporters we often hear the justification that it is safer than alternatives, and there are no real adverse effects. We believe our work has clearly demonstrated that cannabis legalization has measurably impacted the delivery of emergency care in Colorado. However, it is important to put the magnitude of this impact in perspective. Since 2006, more than 2000 Coloradans have died from opioid overdose, and tobacco use-associated healthcare costs in Colorado are almost 2 billion dollars per year. While it is disingenuous to say that cannabis legalization has not impacted emergency medicine in Colorado, it is important to recognize that there are many greater threats to public health and to provide appropriate focus to each of these conditions. A legitimate discussion around the health effects of cannabis in Colorado requires a fair assessment of the risks and benefits by advocates and critics alike.

Continued surveillance on both the positive and negative effects on marijuana legalization, and evidence-based research is needed as more states continue to pass medical and recreational marijuana. The long-term effects of increased availability of high-THC-cannabis are still to be determined. It is critical for public health officials, healthcare providers and legislators, in conjunction with advocates and industry representatives, to work toward regulations aimed at minimizing the public health impact of cannabis legalization on society.
